# AlphaFold-multimer predicts ATG8 protein binding motifs crucial for autophagy research

**DOI:** 10.1371/journal.pbio.3002002

**Published:** 2023-02-08

**Authors:** Hallvard Lauritz Olsvik, Terje Johansen

**Affiliations:** Autophagy Research Group, Department of Medical Biology, University of Tromsø—The Arctic University of Norway, Tromsø, Norway

## Abstract

This Primer explores the implications of a new PLOS Biology study which demonstrates how the AI-based structure prediction tool AlphaFold-multimer can be used to identify sequence motifs that bind to the ATG8 family of proteins central to autophagy.

## Teaser

In this issue of *PLOS Biology*, Ibrahim and colleagues demonstrate how AlphaFold-multimer, an artificial intelligence–based structure prediction tool, can be used to identify sequence motifs binding to the ATG8 family of proteins central to autophagy.

Proteins are structural and executing macromolecules essential for life in all biological systems. Insight into proteins structures is required for detailed mechanistic understanding of how they work and solve different tasks. The ability to predict three-dimensional (3D) protein structures from primary sequence information has therefore been an open research question for more than 50 years. The search for this holy grail of structural bioinformatics has recently led to development of AlphaFold2, an amazing artificial intelligence–based structure prediction tool, developed by scientists from Google DeepMind [1]. How does AlphaFold2 work? Very briefly, it searches sequence databases to find sequences similar to the input, produces a multiple sequence alignment, uses a neural network to extract information, and sends that information to a second neural network that calculates a 3D structure. This is done in an iterative manner. In this issue of *PLOS Biology*, Ibrahim and colleagues demonstrate how AlphaFold2 (AF2)-multimer can be used as an important and powerful new tool to successfully predict so-called LC3 interacting region (LIR) motifs (see below) in proteins involved in autophagy processes [2].

**Fig 1:**
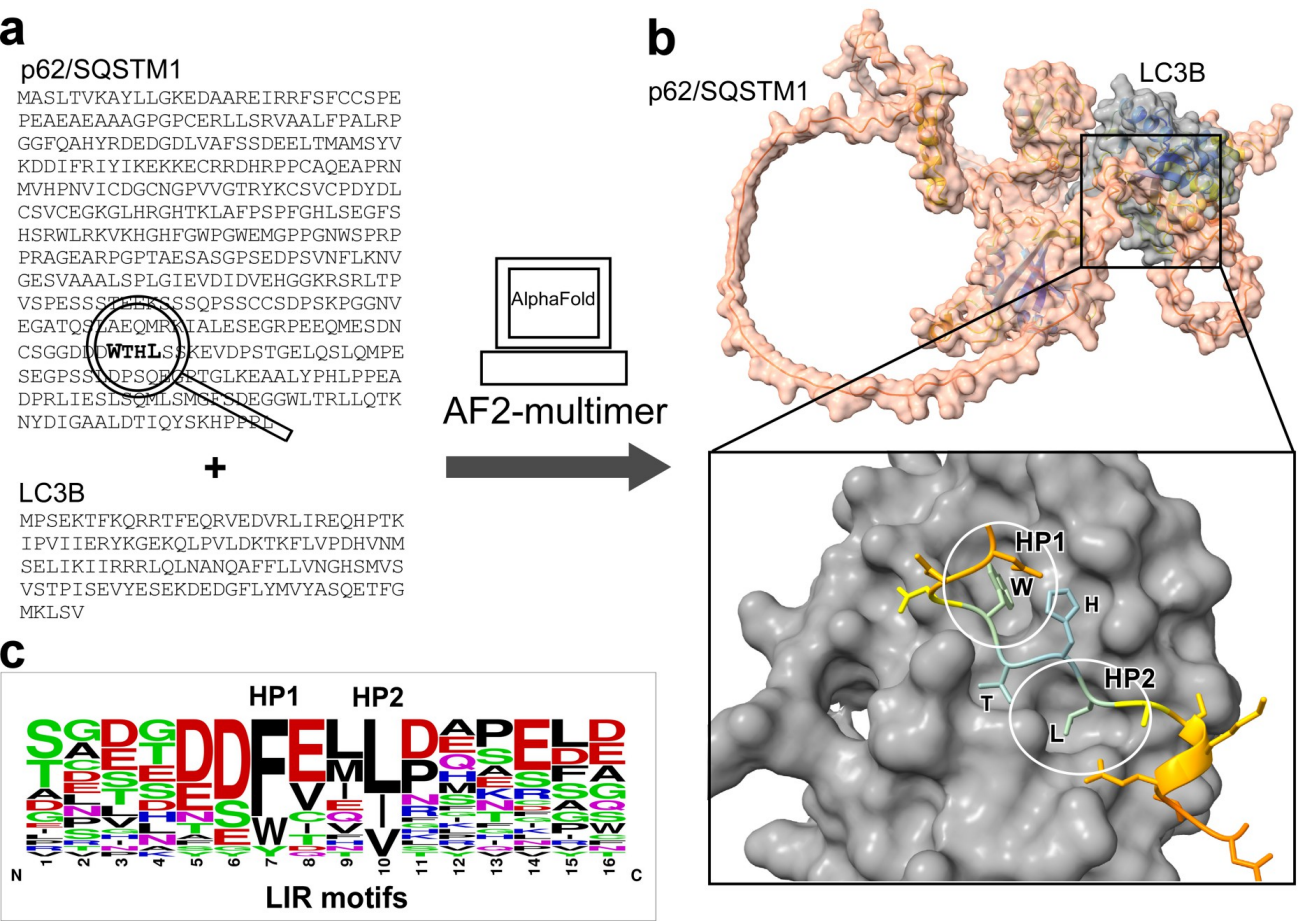
Workflow of LIR predictions using AlphaFold2-multimer. (**a**) The amino acid sequence of the candidate protein (here the human selective autophagy receptor p62/SQSTM1) and the ATG8 protein (here human LC3B) are entered into AlphaFold2-multimer, (**b**) which then provides the predicted structures of the two proteins in complex. They are docked together with the LIR motif positioned into the hydrophobic pockets HP1 and HP2 in the so-called LIR docking site of LC3B as shown in the zoomed inset in b, lower right. This allows both identification of the LIR motif of p62/SQSTM1 (see looking glass in a), which is the already verified LIR motif and a structural model of how the tryptophan (W) and leucine (L) residues of the core LIR motif fits into HP1 and HP2. (**c**) A sequence logo was generated by WebLogo from verified LIR motifs found in 24 autophagy proteins with acidic amino acids indicated in red, basic ones in blue, phosphorylatable serines (S) and threonines (T) in green, and hydrophobic amino acids in black.

Macroautophagy (hereafter autophagy) is an evolutionary conserved lysosomal degradation pathway that maintains homeostasis and executes quality control by selectively removing damaged or surplus macromolecules, organelles, and intracellular pathogens [3]. Autophagy is a multistep process where a double membrane structure is induced, expands, and encapsulates the cargo to be degraded in a closed vesicle, called the autophagosome, which subsequently fuses with lysosomes that digest the cargo. More than 40 conserved autophagy-related (ATG) proteins are involved in this process [4]. Among these is the ATG8 family of ubiquitin-like modifiers represented by three LC3- and three GABARAP family proteins in humans. ATG8 proteins are approximately 120 amino acid long proteins anchored in the membranes of the forming autophagosomes via a C-terminal phosphatidyl ethanolamine lipid moiety. Membrane-conjugated ATG8 proteins have been implicated in cargo sequestration, autophagosome biogenesis and motility, as well as fusion with lysosomes. Their function is mediated through specific interactions with proteins containing LIR motifs [5], which in plants and fungi are often called ATG8 interaction motif (AIM) [6]. A canonical LIR motif is a 10 to 15 amino acids unstructured or β-stranded region usually with a number of negatively charged and/or phosphorylatable residues followed by a core motif of 4 amino acids where the first is a conserved aromatic residue (either W, F, or Y) followed by two much less conserved amino acids and a fourth conserved hydrophobic amino acid (either I, L, or V). The conserved aromatic—and hydrophobic residues bind in two closely spaced hydrophobic pockets (HP1 and HP2) in the ATG8 proteins (**Fig 1**).

LIR motifs were first identified in selective autophagy receptors, which facilitate recruitment of cargo for selective autophagy by binding both to cargo and to LC3 or GABARAP proteins. But also other ATG proteins, adaptors, transport proteins, some protein kinases, and autophagy substrates can bind directly to ATG8 proteins through a LIR motif [5]. Because ATG8 family proteins are central in selective autophagy and in every step of autophagy from autophagosome biogenesis, expansion, transport, and fusion to lysosomes, the LIR/AIM motif has become a focus in autophagy research. To identify and validate candidate LIR motifs in autophagy substrates, potential autophagy receptors or proteins involved in autophagy processes is of crucial importance. How do you identify a novel LIR motif? Hitherto, a standard procedure has been to use a regular expression algorithm based on a consensus sequence curated from previously identified motifs to identify candidate motifs in the linear amino acid sequence of a protein of interest. Most used is the web-based application iLIR [7]. iLIR gives a score for presence of the motif in a potentially unstructured region but does not take into account the 3D structure as such. The predicted LIR motif may be nonfunctional because it is buried or part of a helix with wrong spacing and geometry of the sidechains precluding docking into the HP1 and HP2. Also, the current version of iLIR does not have penalties against selecting candidate motifs with residues within the core LIR that usually are inhibitory to binding such as proline (P), glycine (G), lysine (K), and arginine (R) [8].

As mentioned above, AlphaFold2 has revolutionized prediction of protein structure [1]. The ability to identify amino acid sequence motifs that bind to specific proteins is another central problem in molecular biology. The recent AlphaFold-Multimer (AF2-multimer) now predicts interaction surfaces between two proteins [9]. In this issue of *PLOS Biology*, Ibrahim and colleagues demonstrate how AF2-multimer can be used to successfully predict LIR motifs of both known and unknown LIR-containing proteins and also to identify noncanonical LIR motifs (**Fig 1**) [2]. This is a large step forward that the autophagy research community will benefit from. Ibrahim and colleagues combined protein modelling data from AF2-multimer with phylogenetic analysis of protein sequences and validation of binding in protein–protein interaction assays. In cases where a protein had more than one LIR motif, a smart move was to introduce point mutations into the established LIR motif to search for additional LIR motifs. Searches for LIR motifs was not limited to short stretches of amino acids. Sequences as long as 1,478 residues were used for predicting interactions using AF2-multimer. The procedure works for protein sequences across kingdoms. The authors used it to identify ATG8 binding structures in proteins already shown to bind to ATG8s. The high accuracy of 90% for determining LIR motifs in 33 proteins is extraordinary. The potential of AF2-multimer, and other similar tools like ColabFold [10], does not stop short of autophagy and LIR motifs. It is also of general interest as a strategy and tool to find sequence motifs and/or 3D structures with some degree of conservation binding to specific proteins.

Are these 3D predictions the poor man’s in silico crystal structures? The procedure cannot replace X-ray crystal structures, NMR, or cryo-electron microscopy. However, it is a powerful tool to quickly identify residues that may be important for complex formation and then test these experimentally by site-directed mutagenesis and interaction studies. This will clearly speed up structure activity relation studies in autophagy research and beyond. The computation time to perform AF2-multimer predictions is a limitation and is largely dependent on sequence length. Screening full-length sequences of many proteins for LIRs will be very time consuming. Currently, posttranslational modifications, particularly phosphorylations, that often are important in regulating protein–protein interactions are not handled by AF2-multimer. Future development anticipates the incorporation of the ability to predict effects of phosphorylations and to calculate binding affinities.
